# Improving Engraftment and Immune Reconstitution in Umbilical Cord Blood Transplantation

**DOI:** 10.3389/fimmu.2014.00068

**Published:** 2014-02-24

**Authors:** Robert Danby, Vanderson Rocha

**Affiliations:** ^1^Department of Haematology, Churchill Hospital, Oxford University Hospitals NHS Trust, Oxford, UK; ^2^NHS Blood and Transplant, John Radcliffe Hospital, Oxford, UK; ^3^Eurocord, Hôpital Saint Louis APHP, University Paris VII IUH, Paris, France

**Keywords:** umbilical cord blood, transplantation, hematopoietic stem cells, engraftment, immune reconstitution

## Abstract

Umbilical cord blood (UCB) is an important source of hematopoietic stem cells (HSC) for allogeneic transplantation when HLA-matched sibling and unrelated donors (MUD) are unavailable. Although the overall survival results for UCB transplantation are comparable to the results with MUD, UCB transplants are associated with slow engraftment, delayed immune reconstitution, and increased opportunistic infections. While this may be a consequence of the lower cell dose in UCB grafts, it also reflects the relative immaturity of cord blood. Furthermore, limited cell numbers and the non-availability of donor lymphocyte infusions currently prevent the use of post-transplant cellular immunotherapy to boost donor-derived immunity to treat infections, mixed chimerism, and disease relapse. To further develop UCB transplantation, many strategies to enhance engraftment and immune reconstitution are currently under investigation. This review summarizes our current understanding of engraftment and immune recovery following UCB transplantation and why this differs from allogeneic transplants using other sources of HSC. It also provides a comprehensive overview of promising techniques being used to improve myeloid and lymphoid recovery, including expansion, homing, and delivery of UCB HSC; combined use of UCB with third-party donors; isolation and expansion of natural killer cells, pathogen-specific T cells, and regulatory T cells; methods to protect and/or improve thymopoiesis. As many of these strategies are now in clinical trials, it is anticipated that UCB transplantation will continue to advance, further expanding our understanding of UCB biology and HSC transplantation.

## Introduction

Over the last 25 years, umbilical cord blood (UCB) has become an established alternative source of hematopoietic stem cells (HSC) for use in allogeneic HSC transplantation (1, 2). Due to the lower immunogenicity of UCB grafts and the low rates of graft-versus-host disease (GvHD) compared to bone marrow (BM) and peripheral blood stem cell (PBSC) transplants, less stringent HLA-matching has traditionally been required (3–5). At present, only HLA typing at HLA-A and -B (serological) and HLA-DRB1 (allelic) are commonly used, with mismatches at one or two loci usually being tolerated if sufficient cell doses are transplanted (6). Consequently, UCB transplantation is a potential treatment option for many patients who lack a suitable HLA-matched sibling or unrelated donor. With over 600,000 frozen cord blood units (CBU) stored in cord blood banks worldwide, UCB also has the advantage of being immediately available, avoiding long delays to transplantation, and without any associated risks to the donors (2).

While UCB has increased the applicability of HSC transplantation, UCB transplantation can be associated with delayed engraftment, poor immune reconstitution, and higher rates of infection compared to conventional sources of HSC (4, 5, 7–9). This is due to the quantitative and qualitative differences in the composition of UCB grafts (10). While UCB contains a higher concentration of HSC than adult peripheral blood (PB), each unit contains a one to two log lower total cell dose compared to BM and PBSC harvests (PBSCH). Furthermore, the vast majority of T cells within UCB are antigen-inexperienced, i.e., naïve (CD45RA^+^), being less responsive to allogeneic stimulation, having reduced expression of transcription factors associated with T-cell activation (e.g., nuclear factor of activated T cells, NFAT), and producing lower levels of effector cytokines compared to activated T cells from adult PB (11–13). UCB also contains more immunoregulatory cells, including regulatory T cells (Tregs), with greater potential for expansion and increased suppressive function compared to adult Tregs (13–15). The immaturity of UCB dendritic cells is also associated with lower antigen presenting activity, reduced expression of co-stimulatory molecules (CD80, CD86), reduced cytokine production (TNFα, IL-12), and an inherent ability to induce immune tolerance through peripheral expansion of Tregs (13, 16, 17).

Within this review, the pattern and factors affecting engraftment and immune reconstitution following UCB transplantation will be summarized. We will then provide an overview of current strategies being used to improve engraftment and immune reconstitution following UCB transplantation and potential areas for future research and development (Table 1).

**Table 1:** **Methods to improve engraftment and immune reconstitution in UCB transplantation**.

**1. INCREASING CELL DOSE**
Improved collection and processing of cord blood
Infusion of two cord blood units (double cord blood transplantation)
*Ex vivo* expansion of cord blood HSC/HPC
Infusion of cord blood with third-party donor cells (haploidentical graft)
**2. IMPROVING DELIVERY AND HOMING OF HSC**
Direct intrabone infusion of cord blood
Increased stromal-derived factor-1 (SDF-1) (CXCL12)/CXCR4 interaction (e.g., inhibition of CD26 peptidase)
*Ex vivo* fucosylation of HSC/HPC
**3. IMPROVING SELECTION OF CORD BLOOD UNITS**
Enhanced HLA-matching
Detection of donor specific anti-HLA antibodies
**4. MODIFYING UCB TRANSPLANT REGIMENS**
Using reduced-intensity conditioning
Using T-replete protocols
**5. EXPANDING SPECIFIC CELL POPULATIONS (*EX VIVO* OR *IN VIVO*)**
Natural killer (NK) cells
T cells/pathogen-specific T cells (CMV, EBV, adenovirus)
Regulatory T cells (Tregs)
**6. CO-INFUSING CORD BLOOD WITH ACCESSORY CELLS**
Mesenchymal stem cells (MSC)
**7. IMPROVING THYMOPOIESIS**
Interleukin-7 (IL-7), interleukin-2 (IL-2), and interleukin-15 (IL-15)
Reducing sex steroid hormones (androgen, estrogen)
Growth hormone (GH), insulin-like growth factor 1 (IGF-1)
Keratinocyte growth factor (KGF)
Tyrosine kinase inhibition (sunitinib)
FMS-like tyrosine kinase receptor III ligand (FLT-3-L)
Stem cell factor (SCF)
Inhibition of p53 [pifithrin-β (PFT-β)]

*HSC, hematopoietic stem cells; HPC, hematopoietic progenitor cells; UCB, umbilical cord blood; HLA, human leukocyte antigen*.

## Pattern and Kinetics of Engraftment and Immune Reconstitution Following UCB Transplantation

### Neutrophil and platelet engraftment

Following conditioning [chemotherapy and/or total body irradiation (TBI)] and infusion of the UCB graft, there is an initial period of aplasia during which time the donor UCB HSC and early hematopoietic progenitors engraft, differentiate, and proliferate within the BM environment. The total nucleated cell (TNC) dose and/or CD34^+^ cell dose within the UCB unit(s) (per recipient body weight) are important contributing factors to the probability and rate of neutrophil and platelet engraftment (6, 18). UCB grafts contain a lower TNC dose compared to BM and PBSCH and, therefore, the time to neutrophil engraftment (defined as the first of three consecutive days with a neutrophil count >0.5 × 10^9^/l) is prolonged, with a median time of approximately 30 days for UCB, 21 days for BM harvests, and 14 days for PBSCH (19). Similarly, platelet recovery in UCB transplantation is also prolonged with a median time to engraftment (defined as the first of three consecutive days with an unsupported platelet count >20 × 10^9^/l) ranging from 50 to 100 days. Other important factors influencing engraftment are the degree of HLA-matching, the intensity of the conditioning regimen, and the type of immunosuppression used for GvHD prophylaxis (20).

### Cord blood NK cells: Properties and recovery after UCB transplantation

**Figure 1:**
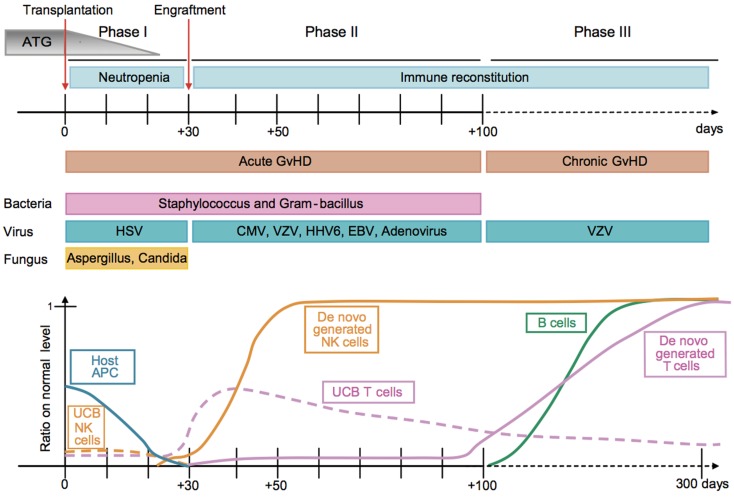
**Kinetics of immune reconstitution and transplant-related complications in children following UCB transplantation**. The post-transplantation period can be divided in three phases. The pre-engraftment period (Phase I, days 0–30) is characterized by general immunosuppression (i.e., depletion of lymphocytes in the recipient by ATG), neutropenia, low platelet counts, and high susceptibility to fungal infections. Host residual APCs progressively disappear during Phase I. Massive proliferation leads NK cells to reach normal levels within 1 month post-transplantation. They may represent 80% of the recipient’s PBLs during the post-engraftment period (Phase II, days 31–100). During Phase II, patients experience high susceptibility to bacterial infections, viral infections (i.e., CMV), and acute GvHD. Some UCB T cells are believed to persist in the recipient and proliferate through HE. This period culminates with the *de novo* generation of T cells through thymopoiesis and the end of the 100-day high-risk window. The late phase (Phase III) is characterized by a higher incidence of VZV infection/reactivation and a progressive reconstitution of B cell and T-cell subsets, which can reach normal levels at 6–9 months post-transplant [figure and legend originally published by Merindol and colleagues (226); used with the permission of H. Soudeyns and the Journal of Leukocyte Biology (Copyright FASEB Office of Publications, Bethesda, MD, USA)].

In keeping with allogeneic HSCT using BM or PBSCH, lymphocyte reconstitution following UCB transplantation typically begins with rapid recovery of natural killer (NK) cells (21) (Figure 1). UCB contains both main NK-cell populations found in PB, i.e., CD16^+^CD56^dim^ and CD16^−^CD56^bright^, although differences in their phenotype, maturity, and function have been reported (22, 23). UCB also contains precursor NK-cell populations, including CD16^+^CD56^−^ cells (24). UCB NK cells have lower cytotoxicity, although their function can be enhanced following interleukin-2 (IL-2), interleukin-7 (IL-7), interleukin-12 (IL-12) or interleukin-15 (IL-15) stimulation (22). They also have lower expression of critical adhesion molecules, e.g., CD2, CD54, and L-selectin, and higher expression of inhibitory receptors including killer-cell immunoglobulin-like receptor (KIR) and NKG2A/CD94 (23). Post-UCB transplant, NK-cell recovery initially occurs through expansion of CD16^−^CD56^bright^ cells with total NK-cell numbers returning to normal values within the first 3 months (21, 25, 26). Beyond this, UCB transplantation is then associated with an increase in both the absolute number and proportion of CD16^+^CD56^dim^ cells compared to BM transplantation (27). Total NK-cell numbers may temporarily exceed the normal range seen in the healthy population, possibly through a compensatory expansion that occurs during periods of profound T-cell lymphopenia (28). This pattern has been reported in both children and adults, following single and double UCB transplants [NK cells are derived from the predominant unit in the case of double UCB transplantation] and can last up to 9 months post-transplantation (21, 25, 26).

### Cord blood T cells: Properties and recovery after UCB transplantation

In allogeneic HSCT, T-cell reconstitution typically occurs in two phases (Figure 1). The first involves early allo-antigen driven homeostatic proliferation of memory T cells, contained either within the graft or, in the setting of T-cell depleted grafts, from residual host T cells escaping pre-transplant conditioning therapy (“thymic-independent”). This, however, produces a restricted T-cell population with limited T-cell receptor (TCR) repertoire against infection. Homeostatic proliferation also occurs faster in CD8^+^ T cells compared to CD4^+^ T cells, producing a reversal of the normal CD4:CD8 T-cell ratio (9, 19). In contrast to BM and PBSCH, UCB mainly contains antigen-inexperienced naïve T cells. Early T-cell reconstitution can therefore only occur via the more stringent *in vivo* priming, activation, and proliferation of the limited naïve T-cell repertoire contained within the graft. The immaturity of UCB T cells is also associated with reduced effector cytokine expression (IFNγ, TNFα) and reduced expression transcription factors involved in T-cell activation (NFAT, STAT4, and T-bet) (11). Consequently, longitudinal studies of immune reconstitution in UCB transplantation have consistently demonstrated profound early T-cell lymphopenia with impaired functional immunity and limited responses to viral infections, in keeping with a primary immune response (9, 28–30).

For long-term effective immune reconstitution with a broad T-cell repertoire, a second T-cell expansion phase is necessary involving thymic production of new naïve T cells (“thymic-dependent”). Hematopoietic progenitors, produced from the engrafted HSC within the BM, enter the thymus to form early T-cell progenitors (ETPs). During T-cell development in the thymus, double positive thymocytes (CD4^+^CD8^+^) are exposed to self-MHC on the thymic cortical epithelial cells. Only those thymocytes that bind to self-MHC with appropriate affinity will be “positively” selected to continue their development into single positive T cells; CD4^+^ T cells interact with MHC Class II molecules, CD8^+^ T cells interact with MHC Class I molecules. Double positive thymocytes that bind too strongly or too weakly to self-MHC undergo apoptosis. As the thymocytes pass through the thymic medulla they are then exposed to self-antigens presented in association with self-MHC molecules. Thymocytes that bind to self-antigens are removed by “negative” selection, thus preventing the production of autoreactive T cells (31). The presence of naïve T cells with markers of recent thymic emigration, i.e., T-cell receptor rearrangement excision DNA circles (TRECs), usually begins around 3–6 months post-UCB transplant (32, 33). However, the timing and effectiveness of thymopoiesis can be impaired by age-related thymic atrophy and/or thymic damage from conditioning therapy and GvHD. Escalon and Komanduri reported a longer delay in the recovery of thymopoiesis, as measured by TREC, in UCB transplantation compared to other HSC sources, possibly due to the limited dose of lymphoid progenitors within the UCB grafts (30). As a consequence, T-cell reconstitution was delayed with a median time to recovery of approximately 9 months for CD8^+^ cytotoxic T cells and 12 months for CD4^+^ helper T cells (25). Similarly, in a retrospective Eurocord analysis of 63 children transplanted with related and unrelated UCB grafts, the median time to T-cell reconstitution was 8 months for CD8^+^ T cells and 12 months for CD4^+^ and total T cells (21). Factors favoring T-cell recovery were HLA-matched UCB, higher nucleated cell dose, and positive recipient cytomegalovirus (CMV) serology prior to transplantation. Conversely, the presence of acute GVHD delayed T-cell recovery. Interestingly, in a recent Eurocord study of children with severe combined immunodeficiency (SCID) transplanted with either UCB (*n* = 74) or haploidentical grafts (*n* = 175), there were no significant difference in T-cell recovery (total T cells and CD4^+^ T cells) between the groups at any time, although the UCB transplant recipients had significantly faster recovery of total lymphocyte counts (34).

Over the last few years, the use of double cord blood transplants has significantly increased, mainly in adults following reduced-intensity conditioning (RIC) regimens. In this setting, although two CBUs are initially transplanted, only one provides prolonged engraftment, i.e., the “predominant” unit. Few studies have reported data on T-cell recovery after double CBT. Ruggeri and colleagues reported outcomes, infection rates, and immune reconstitution after 35 double UCB transplants in recipients with high-risk hematological diseases (25). Lymphocyte subset analyses were performed at 3, 6, 9, and 12 months post-transplant and demonstrated reduced T and B cell counts until 9 months. Recovery of thymopoiesis, as measured by TRECs, was also impaired until 9 months post-transplant. Somers and colleagues also analyzed engraftment kinetics in leukocyte subsets following non-myeloablative double UCB transplants (26). CD4^+^ T cells, CD8^+^ T cells, and NK cells all showed early engraftment predominance by day 11, followed by predominance in myeloid cells by day 18. Based upon these findings, it is proposed that T cells and/or NK cells from the predominant unit may elicit an early immune response against the second unit.

### Cord blood B cells: Properties and recovery after UCB transplantation

B cell reconstitution occurs over the first 6 months post-UCB transplantation, although full recovery of immunoglobulins takes longer (Figure 1). In 63 children given related or unrelated UCB transplants, the median time to B cell recovery was 6 months (21). PB CD19^+^CD28^high^CD38^high^ transitional B cells are first detectable at 1–2 months post-transplant. Over the next 3–6 months, total B cell numbers gradually rise with an associated increase in the proportion of mature CD19^+^CD28^int^CD38^int^ mature B cells to approximately 90% by 9 months (35). As reported with NK cells, total B cell numbers may also expand above the normal range during the period of T-cell lymphopenia before returning to normal values when T-cell recovery occurs (28). At the same time, levels of immunoglobulins to commonly encountered antigens typically rise to normal levels by the end of the first year. Recovery of B cell function, as measured by the time to discontinuation of intravenous immunoglobulin replacement therapy, was compared following UCB (*n* = 74) and haploidentical transplants (*n* = 175) in children with SCID (34). At 3 years after transplantation, 45% of the UCB transplant recipients had discontinued immunoglobulins, compared with 31% of the haploidentical recipients (*P* = 0.02). Other factors associated with improved B cell function were the absence of pre-transplantation infections and use of myeloablative conditioning (MAC).

## Implications of Delayed Immune Reconstitution Following UCB Transplantation

As a direct consequence of delayed immune reconstitution, UCB transplantation is associated with a significant risk of opportunistic infections, particularly viral infections including CMV, varicella zoster virus (VZV), Epstein–Barr virus (EBV), adenovirus (ADV), human herpesvirus-6 (HHV-6), and BK virus (Figure 1). In a recent retrospective review of 332 UCB transplants performed at the University of Minnesota between 1994 and 2007, 51% of CMV-seropositive patients (*n* = 92/180) experienced CMV reactivation, with a median time to reactivation of 40 days (range, 9–95 days) (36). CMV infection was infrequent in CMV-seronegative recipients (1.3%, *n* = 2/152). Neither pre-transplant CMV-seropositive status nor CMV reactivation was associated with increased transplant-related mortality (TRM). However, in the 14% (*n* = 25/180) of CMV seropositive patients that developed CMV disease (16 respiratory; 6 gastrointestinal; 3 multi-organ), TRM was significantly higher and overall survival reduced. It is hypothesized that the high incidence of CMV reactivation/infection post-UCB transplantation is related to impaired CMV-specific T-cell responses. In a study of T-cell reconstitution in 28 pediatric UCB transplants using MAC, CD4^+^ and CD8^+^ T cells were persistently low for the first 3 months post-transplant (37). By 3 months, only two patients had developed CMV-specific T cells and only one patient had VZV-specific T cells, as measured by antigen-specific IFNγ ELISPOTS. CMV and VZV responses developed over the next 3 years in 30% and 40% of the recipients, respectively. However, development of CMV or VZV disease was not necessary to acquire these responses and no subject developed infections once satisfactory responses had been attained (>150 spot forming units/10^6^ PBMC). More recently, McGoldrick and colleagues were able to demonstrate CMV-specific polyclonal CD4^+^ and CD8^+^ T-cell responses from the donor UCB graft by day 42 but at insufficient numbers to be able to control viral reactivation (38). The lack of sufficient CMV-specific CD8^+^ T cells to control CMV reactivation appears to be due to insufficient *in vivo* expansion, either due to immunosuppressive therapy and/or early deficiency of CD4^+^ T cells. Later control of CMV reactivation was due to improved function of the T cells primed early after transplant rather than *de novo* responses from the thymic-dependent pathway.

In a retrospective study of EBV reactivation, 4.5% (*n* = 15/335) patients receiving an UCB transplant between 1994 and 2005 developed EBV related complications (4 viremia; 11 PTLD) (39). More recently, in a retrospective Eurocord study of 175 UCB transplants in which EBV viral load was monitored by RT-PCR during the first 3 months post-transplant, 24 patients had EBV reactivation with a median time of 86 days (range, 14 days to 2.7 years) (40). The cumulative incidence of EBV reactivation by 100 days was 8% (*n* = 15) and four patients developed EBV-PTLD (cumulative incidence 2%) at a median time of 73 days (range, 63–80 days) (median of 28 days after the first positive EBV RT-PCR result). Other viral infections also have a higher prevalence in UCB transplants compared to BM and PBSCH. VZV reactivation is more frequent in UCB transplantation than BM transplants [RR 2.27 (95% confidence interval (CI), 1.18–4.34), *P* = 0.013], as is VZV dissemination (41). HHV-6 is a double stranded DNA virus that can reactivate following HSCT and may cause encephalitis and/or pneumonitis. In a recent meta-analysis, the prevalence of HHV-6 reactivation and HHV-6 encephalitis was higher in patients receiving UCB than other stem cell sources (72 vs. 37%, *P* < 0.0001; 8 vs. 0.5%, *P* < 0.0001, respectively) (42). BK virus is another double stranded DNA virus, belonging to the polyomavirus family, and has been associated with hemorrhagic cystitis post-HSCT. A retrospective analysis of 209 HSCT demonstrated that both BK PCR positivity pre-transplant and receiving an UCB or haploidentical graft with MAC was associated with a significantly higher risk of developing hemorrhagic cystitis (43).

Opportunistic infections are associated with significant mortality following UCB transplantation and are a major contributing factor in >40% of deaths following UCB transplants, particularly in the first 3 months (5, 7). In a review of 330 single unit UCB transplants at Duke University, 110 patients died within the first 6 months, of which 58% were due to opportunistic infection (viral, fungal, or protozoal) (9). Twenty-two patients died from ADV and 12 from CMV infection. In a Grupo Español de Trasplante Hematopoyético (GETH) study of 192 consecutive adult unrelated allogeneic HSCT, the 100-day and 3-year infection-related mortality (IRM) for UCB transplants (*n* = 48) was 30% (95% CI, 10–40%) and 40% (95% CI, 12–58%), respectively (44). However, although IRM post-UCB transplantation remains a concern, it is unclear whether UCB transplantation has a higher proportion of deaths due to infection compared to other forms of HSCT. In an IBMTR comparison of unrelated donor transplants for leukemia, Laughlin and colleagues reported the proportion of deaths due to infection within 100 days as 45, 21, and 24% (*P* = 0.01) for UCB (*n* = 150), HLA-matched marrow (*n* = 367), and HLA-mismatched marrow (*n* = 83), respectively (8). However, Rocha and colleagues observed that a similar proportion of transplant deaths were due to infection when comparing cord blood (42%, 18/62) to unrelated BM (41%, 41/320) (5). In the Spanish GETH study, although UCB transplants had a higher risk of severe infection compared to BM/PBSC transplants (85 vs. 69%, *P* < 0.01), the 100-day IRM (30 vs. 28 vs. 22%; *P* = 0.2) and 3-year IRM (40 vs. 42 vs. 38%, *P* = 0.5) were not significantly different (44). Likewise, in a study of 136 pediatric unrelated donor transplants, the proportion of patients in which infection was causal or contributing toward death was not significantly different (BM 36%, T-cell depleted BM 33%, UCB 30%) (45).

## Improving Engraftment and Immune Reconstitution Following UCB Transplantation

### Use of double cord blood transplantation

In UCB transplantation, the cell dose (TNC dose and/or CD34^+^ dose) and HLA-matching of the graft are important factors for successful engraftment in both pediatric and adult patients (3, 5, 7). Therefore, in 2009, Eurocord published recommendations for selection of CBUs for transplantation (6). In summary, when a single CBU (6/6 or 5/6 HLA-matched) does not contain sufficient number of cells (TNC >2.5 × 10^7^/kg upon freezing; >2.0 × 10^7^/kg on thawing), double cord blood transplantation should be considered, aiming for a combined TNC dose >3.0 × 10^7^/kg. Even higher doses are recommended if the single CBU is only 4/6 HLA-matched (TNC >3.5 × 10^7^/kg upon freezing; >2.5 × 10^7^/kg on thawing for malignant disorders; TNC >4.0 × 10^7^/kg upon freezing; >3.5 × 10^7^/kg on thawing for non-malignant disorders). UCB transplants using two units from different donors were first reported in 2001 by the Minneapolis group in an attempt to increase cell dose infused in adults and older children (46). Both units contribute to early engraftment, although eventually, one unit predominates (47). In an analysis of 23 double UCB transplants following MAC, hematopoiesis was observed from a single donor in 76% patients at day 21 and 100% patients by day 100 (48). Likewise, on review of 81 patients with sustained chimerism after receiving a double UCB transplant using a non-myeloablative regimen, single donor chimerism was detectable in 57, 81, and 100% patients at day 21, 100, and 365, respectively (47). Double UCB transplants show high rates of engraftment (85–100%) with the median time to neutrophil engraftment ranging from 9 to 33 days depending on the conditioning regimen and/or the use of granulocyte colony stimulating factor (GCSF) (26, 47, 49). Interestingly though, a significant difference in the rate of engraftment has not been demonstrated between patients receiving one or two CBUs (47, 50). However, a lower relapse risk was found in patients receiving two CBUs for acute leukemia (CR1/CR2), possibly through an enhanced graft-versus-leukemia (GvL) effect (50). Recently, Ruggeri and colleagues reported the outcomes of 35 double cord blood transplants in recipients with high-risk hematological diseases (25). The cumulative incidence of neutrophil recovery was 86%, acute GvHD 47%, and first viral infection 92%. Immune recovery was delayed with reduced T and B cell counts and compensatory expansion of NK-cells observed until 9 months post-transplant, followed by the appearance of new thymic precursors.

### Cord blood expansion

An alternative approach being used to increase cell dose in UCB transplantation is *ex vivo* expansion of cord blood. As well as increasing the total number of HSC cells for long-term engraftment, this may also increase the number of committed progenitors to reduce the initial period of neutropenia. *Ex vivo* expanded CB can then be given alone or in combination with an unmanipulated unit. In this setting, although the expanded unit improves early hematopoietic recovery, it is the unmanipulated unit that usually provides long-term engraftment (51). UCB expansion has been achieved using several different methods. The first is liquid culture in which isolated CD34^+^ or CD133^+^ HSC are expanded in the presence of selected cytokines and growth factors, including stem cell factor (SCF), thrombopoietin (TPO), GCSF, and/or FMS-like tyrosine kinase 3 ligand (FLT-3-L) (52, 53). The optimal milieu of cytokines and growth factors remains uncertain but several groups have shown improved expansion by the addition of IL-3 and/or IL-6 (54). Shpall and colleagues performed a feasibility study in which CD34^+^ cells were isolated from a fraction (40–60%) of the CBU and expanded in liquid culture with SCF, GCSF, TPO, and megakaryocyte growth and differentiation factor (52). The remainder of the unit was then infused with the expanded cells following MAC. The median TNC dose infused was 0.99 × 10^7^/kg and the median time to engraftment was 28 days (range, 15–49 days) for neutrophils and 106 days (range, 38–345 days) for platelets. Using a modification to this approach, a Phase I/II trial was performed in which CD133^+^ cells were isolated from a portion of the CBU and expanded in liquid cultures with SCF, FLT-3-L, IL-6, TPO, and the copper chelator TEPA (55). The median TNC fold expansion was 219 (range, 2–260). Both expanded and unexpanded cells were infused with a median TNC of 1.8 × 10^7^/kg. Nine of the 10 patients engrafted with a median time to neutrophil and platelet engraftment of 30 days (range, 16–46 days) and 48 days (range, 35–105 days), respectively. Delaney and colleagues reported results from a Phase I trial using an immobilized Notch ligand Delta-1 in addition to SCF, FLT-3-L, TPO, IL-3, and IL-6 (56). Ten patients with high-risk leukemia were treated with a myeloablative double UCB transplant in which one unit was expanded using this protocol. The average fold expansion was 562 (range, 146–1496) for TNC and 164 (range, 41–471) for CD34^+^ cells. Nine of the 10 patients engrafted with a median time to neutrophil engraftment of 16 days (range, 7–34 days). However, in contrast to other reported studies, there was a predominance for donor CD33^+^ and CD14^+^ cell engraftment from the expanded unit.

The second expansion method uses co-culture with a supporting network of mesenchymal stromal cells to provide a hematopoietic microenvironment that supports HSC proliferation (57). de Lima and colleagues reported the results of 31 patients receiving 2 CBUs, 1 of which was expanded *ex vivo* with mesenchymal stem cells (MSC). This *ex vivo* culture system expanded TNC and CD34^+^ cells by a median factor of 12.2 and 30.1, respectively, and the median TNC dose infused was 8.34 × 10^7^/kg (51). Of the 24 patients who received *ex vivo* expanded cells, 23 achieved neutrophil engraftment, at a median time of 15 days (range, 9–42 days), and 18 had sustained platelet engraftment, at a median time of 42 days (range, 15–62 days). Both compared favorably to 80 CIBMTR historical controls that received unmanipulated double UCB transplants only [neutrophil engraftment 24 days (range, 12–52 days), *P* < 0.001; platelet engraftment 49 days (range, 18–264 days), *P* = 0.03]. In addition, while the expanded CBU improved early hematopoietic recovery, in all cases, the unmanipulated unit provided long-term engraftment.

HSC expansion has also been achieved using a continuous perfusion culture system in which cells are supplied with fresh culture media and gaseous exchange (58, 59). In a Phase I study, Jaroscak and colleagues expanded a portion of a CBU using a continuous perfusion culture device and infused these expanded cells 12 days after the remainder of the original unit (58). The median fold increase in TNC was 2.4 (range, 1.0–8.5). Twenty-one of the 26 patients attained neutrophil engraftment with a median time of 22 days (range, 13–40 days). The median time for platelet engraftment was 71 days (range, 39–139 days; *n* = 16). Ongoing early phase clinical trials of all three methods are in progress.

### Combined use of UCB with third-party donor

Another strategy being used to improve engraftment following UCB transplantation is the combined use of cord blood and haploidentical transplants (60, 61). While the haploidentical graft provides early engraftment, it is the UCB graft that usually provides long-term engraftment. Sebrango and colleagues reported the results of 55 combined UCB/haploidentical transplants for high-risk myeloproliferative and lymphoproliferative disorders (60). The maximum cumulative incidence of neutrophil and platelet engraftment was 96 and 78% with a median time to recovery of 10 and 32 days, respectively. Full UCB chimerism was achieved in 50 patients [cumulative incidence 91% (95% CI, 84–99%)] with a median time of 57 days (range, 11–186 days). Immune reconstitution analysis showed NK-cell recovery occurred within 3 months, at a time when patients had dual UCB/haploidentical chimerism. However, B and T cells recovered by 6 months and 1 year, respectively when full UCB chimerism had been attained. Liu and colleagues transplanted 45 patients using a RIC regimen with an unrelated UCB graft and CD34^+^ selected cells from a haploidentical donor (61). The cumulative incidence of neutrophil engraftment was 95% at day 50 with a median time to recovery of 11 days. The cumulative incidence of platelet engraftment was 83% at day 100 with a median time to recovery of 19 days. The median percentage of PB cells of UCB origin was 10, 78, and 95% at day 30, 100, and 180, respectively. Conversely, the median percentage of PB cells from the haploidentical graft was 86, 22, and 2% at the corresponding times. The cumulative incidence of acute and chronic GvHD was 25 and 5%, respectively, with non-relapse mortality (NRM) at 1 year 38%, relapse 30%, and overall survival 55%.

### Improving delivery and homing of UCB HSC

To improve HSC engraftment following UCB transplantation, direct intrabone infusion of cord blood cells is currently being investigated. In a Phase I/II study, 32 consecutive patients with acute leukemia received UCB transplants with intrabone infusion between 2006 and 2008 (62). No complications occurred during administration. The median time to neutrophil and platelet engraftment was 23 days (range, 14–44 days; *n* = 28) and 36 days (range, 16–64 days; *n* = 27), respectively, and all engrafted patients showed full donor chimerism from day 60 onward. Sixteen patients were alive and in remission with a median follow-up of 13 months. Okada and colleagues demonstrated in a Phase I study that intrabone infusion of unwashed cord blood following a RIC regimen was also well tolerated (63). In 10 patients, there were no injection related complications and the median time to neutrophil recovery was 17 days. Saglio and colleagues showed that intrabone injection was also well tolerated in children (64). In a recent Eurocord retrospective comparison of single unit intrabone UCB transplants (*n* = 87) with double unit intravenous UCB transplants (*n* = 149), intrabone infusion was associated with improved neutrophil engraftment by day 30 (76 vs. 62%, *P* = 0.014) and improved platelet engraftment by day 180 (74 vs. 64%, *P* = 0.003). Intrabone infusion was also associated with a lower incidence of acute GvHD and had a trend toward improved disease-free survival (DFS) (65). These results are encouraging and further clinical trials are currently ongoing to evaluate engraftment kinetics and immune reconstitution following intrabone infusion (66).

Factors that promote homing of UCB HSC to the BM niche may also improve engraftment. Stromal-derived factor-1 (SDF-1) (CXCL12) is produced by BM stromal cells and binds to its receptor, CXCR4, on the surface of HSC, pre-B lymphocytes, and T cells. SDF-1 levels are increased following HSCT conditioning and the infused HSC follow the SDF-1 gradient toward the BM niche. Once engrafted, SDF-1 may also promote HSC proliferation and survival (67, 68). Many factors increase the sensitivity of CXCR4 on HSC to SDF-1, including complement (C3a), hyaluronic acid, VCAM-1, fibrinogen, and thrombin. Therefore, *ex vivo* priming of UCB HSC with these molecules may promote homing and engraftment of the HSC (69, 70). Inhibition of the membrane bound extracellular peptidase (CD26), which cleaves SDF-1, also enhances long-term engraftment in UCB CD34^+^ cells in NOD/SCID/beta 2 microglobulin null mice (71, 72). Fucosylation of ligands expressed on HSC is also required for their interaction with selectins expressed in the BM microvasculature. In NOD-SCID interleukin-2Rγ (null) mice, Robinson and colleagues demonstrated that only fucosylated UCB CD34^+^ were responsible for engraftment and that *ex vivo* fucosylation improved UCB engraftment rates (73). All these pre-clinical studies show encouraging results and, as such, further investigation is warranted to determine whether these techniques can improve HSC engraftment in clinical UCB transplantation.

### Enhanced HLA-matching and detection of HLA antibodies

Until recently, CBUs for transplantation were selected using cell dose (TNC and/or CD34^+^) and HLA-matching at HLA-A and -B (antigen) and HLA-DRB1 (allele). However, the importance of enhanced HLA-matching has now been recognized. In 2011, Eapen and colleagues retrospectively reviewed the results from 803 single UCB transplants (leukemia/MDS), analyzing the impact of HLA typing at HLA-A, -B, and -C (intermediate resolution) and HLA-DRB1 (allelic) (74). Neutrophil recovery (day 28) was inferior in UCB transplants mismatched at three or more HLA-loci (70, 64, 64, 54, and 44% with zero, one, two, three, and four mismatches, respectively). In addition, TRM was higher when CBU were mismatched at two or more HLA-loci [HR 3.27 (*P* = 0.006); HR 3.34 (*P* = 0.005); HR 3.51 (*P* = 0.006) for two, three, and four mismatches]. Compared to fully matched transplants (8/8), CBU mismatched at HLA-C had higher TRM [HR 3.97 (*P* = 0.018)]. TRM was also higher in UCB transplants with a single HLA-mismatch at HLA-A, -B, or -DRBI and mismatched at HLA-C compared to transplants with a single HLA-mismatch at HLA-A, -B, or -DRBI but matched for HLA-C [HR 1.70 (*P* = 0.03)]. Additional matching for HLA-C was therefore recommended. More recently, a joint CIBMTR and Eurocord study analyzed the effect of high resolution (allele) typing at HLA-A, -B, -C, and -DRB1 on the outcomes of 1658 MAC single UCB transplants (75). Neutrophil recovery (day 28) was inferior in transplants mismatched at three or more alleles compared to fully matched CB [odds ratio (OR) 0.56 (95% CI, 0.36–0.88) *P* = 0.01; OR 0.55 (95% CI, 0.34–0.88) *P* = 0.01; OR 0.45 (95% CI, 0.25–0.82), *P* = 0.009 for three, four, and five allelic mismatches]. NRM was also associated with the degree of HLA-mismatching. Single HLA-allele mismatches at HLA-A, -C, or -DRB1 had higher NRM [HR 3.05 (1.52–6.14), *P* = 0.02; HR 3.04 (95% CI, 1.28–7.20), *P* = 0.01; HR 2.93 (95% CI, 1.38–6.25), *P* = 0.005, respectively]. Importantly, CBUs with TNC <3.0 × 10^7^/kg were associated with significantly higher NRM, independent of HLA-matching. Therefore, Eapen and colleagues proposed that single UCB transplants should have a minimum TNC dose of 3.0 × 10^7^/kg. The best HLA-allele matched CBU should then be selected. However, units with three or more HLA-allele mismatches should only be used with caution due to increased graft failure and higher NRM.

In UCB transplantation, screening for donor specific anti-HLA antibodies (DSA) should also be considered. In a retrospective analysis of 386 MAC single UCB transplants performed for hematological malignancies, 89 patients had anti-HLA antibodies, 20 with specificity against the CBU (76). In multivariate analysis, neutrophil and platelet recovery were significantly worse in these 20 patients compared to the antibody negative group [RR 0.23 (0.09–0.56), *P* = 0.001; RR 0.31 (95% CI, 0.12–0.81), *P* = 0.02, respectively]. Similarly, in 73 double UCB transplants, the presence of DSA was associated with increased graft failure (5.5 vs. 18.2 vs. 57.1% for none, single, or dual DSA positivity; *P* = 0.0001) and a longer median time to neutrophil recovery [29 days (any DSA) vs. 21 days (no DSA), *P* = 0.04] (77). More recently, a retrospective Eurocord analysis on the impact of DSA in 294 RIC UCB transplants was performed. 21% recipients had anti-HLA antibodies of which 14 (5%) had donor specificity. Day 60 neutrophil engraftment (44 vs. 81%, *P* = 0.006) and 1 year TRM (46 vs. 32%, *P* = 0.06) were inferior in the presence of DSA (78). In 70 children receiving single UCB transplants, the presence of antibodies to major-histocompatibility-complex Class I-related chain A antigen (MICA) was also associated with delayed platelet engraftment [HR 4.2 (95% CI, 1.02–17.08), *P* = 0.04] (79). While not all studies have found this association between DSA and engraftment, possibly due to use of lower thresholds for DSA detection, it is recommended that potential recipients be screened for anti-HLA antibodies before UCB transplantation (79, 80). CBUs for which the recipient has high levels of DSA should then be avoided. The full implication of DSA at lower levels remains less clear and further evaluation is required.

### Reduced-intensity conditioning and T-replete transplants

The use of RIC regimens in all forms of allogeneic HSCT has increased over the last decade. RIC regimens are less myeloablative but provide sufficient immunosuppression to allow donor engraftment. Disease eradication is then dependent upon the donor-derived T cells recognizing residual tumor as “non-self,” producing an immune mediated graft-versus-tumor (GvT) response (81). RIC regimens have less toxicity and lower TRM, therefore allowing transplantation to be performed in older patients and/or in those with other significant co-morbidities that would otherwise prevent HSCT. RIC regimens are now commonly used in both pediatric and adult UCB transplants (47, 82). However, immune reconstitution following RIC UCB transplantation has not been extensively reported. Geyer and colleagues monitored immune subset recovery in 88 consecutive UCB transplants of which 49 had MAC and 39 had RIC (83). In this series, no significant difference was observed in T, B, or NK-cell recovery or immunoglobulin reconstitution, although the two groups were not evenly matched for other potential confounding factors, e.g., disease type and status at transplant.

To improve engraftment and limit GvHD, many UCB transplant regimens also use *in vivo* T-cell depletion with anti-thymocyte globulin (ATG). ATG is a polyclonal antibody, prepared in rabbits or horses, raised against thymocyte antigens (84). Although infused into the recipient pre-transplant, the half-life of ATG is such that it reduces T cells in both the recipient and infused grafts, producing profound T-cell depletion and delaying immune reconstitution. Chiesa and colleagues recently published a study measuring early immune reconstitution in 30 pediatric UCB transplants without *in vivo* T-cell depletion (85). In keeping with T-depleted transplants, NK-cell recovery was rapid with a median time to recovery of 1 month (1–3 months). However, in contrast to ATG-based protocols, T-cell recovery was faster with a rapid “thymic-independent” expansion, broader T-cell repertoire with virus-specific responses, and rapid conversion from naïve to central memory phenotype. In particular, CD4^+^ T-cell recovery was faster with a median count of 0.6 × 10^9^/l at 2 months post-UCB transplant. Although CD8^+^ T-cell recovery was delayed, it was still faster than previously reported for T-depleted UCB transplants. Similarly, B cell reconstitution was also faster. Twenty-nine of the 30 patients engrafted with the median time to neutrophil engraftment of 22 days (range, 13–38 days) and platelet engraftment of 42 days (range, 17–123 days). The cumulative incidence of grade II–IV acute GvHD was 50% but chronic GvHD was relatively low at 14%. In a similar review in adult patients, T-cell reconstitution was measured in 72 double UCB transplants (52 myeloablative and 20 non-myeloablative) without ATG (86). In this group, the median CD4^+^ T-cell count was 0.27 × 10^9^/l at 4 months. Again, CD4^+^ T-cell recovery was faster than that seen in similar series of single and/or double UCB transplants using ATG. Four patients had graft failure and the median time to neutrophil engraftment was 23 days (range, 11–43 days) in the myeloablative group and 11 days (range, 7–36 days) in the non-myeloablative group. The cumulative incidence of acute GvHD at day 100 was 43% (95% CI, 33–56%) and survival at 1 year was 68% (95% CI, 57–79%). Therefore, both studies show promising results with potentially improved immune reconstitution compared to T-depleted UCB transplants although this has to be balanced against the risk of GvHD. In addition, as recognized by the authors, the limitations of comparing results from different retrospective series must be considered and further studies will be necessary.

### Increasing specific cell populations

#### NK cells

Natural killer cells have an important role in early immunity against infection and the GvL response following HSCT. NK-cells expressing the activation receptor, NKG2C, and producing IFNγ rapidly expand following acute CMV reactivation, reflecting a primary NK-cell response (87). These cells persist long after viral clearance and develop a mature phenotype being CD56^dim^ with high KIR expression and reduced expression of NKG2A. In HLA-haploidentical transplants, donor-versus-recipient NK-cell alloreactivity also protects against rejection, GvHD, and acute myeloid leukemia (AML) relapse (88, 89). NK-cell alloreactivity arises from a mismatch between the inhibitory receptors for self-MHC Class I molecules on NK cells and MHC Class I antigens on recipient cells (90). Furthermore, KIR ligand incompatibility in the GvH direction is an independent predictor of overall survival in AML (89). In light of these observations, NK-cell immunotherapy has been used as consolidation treatment in high-risk AML patients and following leukemic relapse after haploidentical HSCT (91, 92).

In relation to UCB transplantation, isolation and infusion of NK cells to improve immune recovery and/or treat relapse has proven difficult due to the low number of cells available in CBUs. To resolve this issue, several groups have developed *ex vivo* NK-cell expansion protocols using UCB (93–96). Beck used Notch receptor ligand Delta-4 to differentiate and expand UCB CD34^+^ cells into NK cells (93). Most were CD16^−^CD56^bright^ and did not express inhibitory receptors that bind Class I MHC (NKG2A, KIR) but did express activating NK receptors (NKG2D) required for cytotoxicity against leukemia cells. Tanaka and colleagues expanded NK cells from unmanipulated UCB cells (1 × 10^6^) using Tacrolimus and low molecular weight heparin without feeder cells (95). This good manufacturing practice (GMP) compliant method produced 40 × 10^6^ NK cells with high levels of stimulatory NK-cell receptors (NKG2C, NKG2D, NKp44). Recently, the same group also reported that the tyrosine kinase inhibitor, Dasatinib, can enhance expansion of NK cells from unseparated UCB and, therefore, could be potentially used to expand NK cells both *ex vivo* and *in vivo* (97). Use of NK cells in UCB transplantation is currently being investigated in clinical trials (98).

#### T cells

In view of the prolonged T-cell lymphopenia and high rates of viral infection observed post-HSCT, there has been much interest in using adoptively transferred viral-specific mature T cells to improve immune reconstitution and prevent/treat infection. Pathogen-specific T cells can be expanded *ex vivo* from small quantities (<2.5%) of GCSF-mobilized PBSCH and have been used in Phase I/II trials or in selected cases of refractory CMV or EBV infection (99–102). *In vitro* techniques are also being developed to detect, enrich, and produce multiple virus-specific T cells allowing simultaneous adoptive transfer of T cells directed against CMV, EBV, BKV, and ADV (103, 104). For UCB transplantation, however, this strategy has proved much more difficult due to the small number of cells available and because the majority of UCB T cells are antigen naive. Initial strategies employed *ex vivo* polyclonal expansion of all UCB T cells from an aliquot of the CBU using anti-CD3/CD28 coated Dynabeads and IL-2 (105, 106). Although expansion and maturation of the T cells was possible, significant apoptosis of CD4^+^ T cells occurred and there was a reversal of the normal CD4:CD8 ratio. Addition of IL-7 to the cultures reduced apoptosis, increased proliferation to an average fold expansion of 165, and promoted functional maturation (107). Importantly, the expanded cells lacked alloreactivity against allogeneic cells but could be primed against leukemia cells to generate tumor-specific cytotoxic T cells. Clinical trials of these *ex vivo* expanded T-cell products will be necessary. In relation to pathogen-specific T cells, Park and colleagues first demonstrated that CMV-specific T cells could be produced from UCB by isolation of T cells, priming with IL-7 and IL-12 and stimulation with monocytes and dendritic cells containing CMV antigen (108). More recently, Hanley and colleagues further developed these techniques to produce GMP-compliant cytotoxic T cells against CMV, EBV, and ADV (109). In the first expansion phase, UCB derived T cells are stimulated with UCB dendritic cells transduced with adenoviral vector containing the CMV antigen, pp65, in presence of IL-7, IL-12, and IL-15. A second stimulation is then performed using EBV-transformed B cells. This method only requires 20% of the CBU and can produce 150 × 10^8^ viral specific cells that lyse antigen-pulsed targets and release effector cytokines in response to antigen stimulation. Phase I/II trials using the pathogen-specific expanded UCB T cells are currently in recruitment (110).

#### Regulatory T cells

The fundamental principle of the human immune system is to protect the body from harmful pathogens (“non-self”) while being unresponsive to self-antigens (“self-tolerance”). In addition to the passive central mechanisms of tolerance (“positive” and “negative” selection in the thymus), tolerance is also maintained by peripheral immune mechanisms. Several cells (“suppressors”) can suppress autoreactive clones through dominant mechanisms. Of these, Tregs are arguably the most understood. In 1995, Sakaguchi and colleagues described a population of CD4^+^ T cells expressing the IL-2 receptor alpha chain (CD25) (111). When CD4^+^CD25^−^ cells, from BALB/c nu/^+^ mice were transferred into BALB/b nu/nu mice they induced a widespread autoimmune disease that could be prevented by co-transfer of donor CD4^+^CD25^+^ cells. The CD4^+^CD25^+^ T cells subsequently became known as Tregs and, in 2001, human CD4^+^CD25^+^ Tregs were described (112, 113). In 2003, the transcription factor forkhead box P3 (Foxp3) was found to be specifically expressed in Tregs and is thought to be the master regulator of Treg differentiation and function (114, 115). Scurfy mice have functional mutations in *Foxp3* producing a deficiency of Tregs and a severe multi-systemic autoimmune disorder (114, 116, 117). Conversely, ectopic expression of Foxp3 in murine naïve CD4^+^ T cells produces suppressive function and expression of other Treg phenotypic markers, e.g., cytotoxic T lymphocyte antigen 4 (CTLA-4) (114, 115). FOXP3 is also found in human Tregs and mutations in *FOXP3* cause the IPEX syndrome (Immune dysregulation, Polyendocrinopathy, Enteropathy, X-linked) (118, 119).

Tregs induce peripheral tolerance by inhibiting the proliferation and cytokine secretion of T, B, NK, NKT, and antigen presenting cells. Many functional mechanisms have been proposed although the contribution of each one *in vivo* remains unclear (120). Cell-contact independent mechanisms include sequestration of IL-2 and the production of inhibitory cytokines such as IL-10 and IL-35. As Tregs express high levels of CD25, Tregs may preferentially absorb IL-2, causing Bim-mediated apoptosis in effector cells due to relative IL-2 deficiency (121). IL-10 [human cytokine synthesis inhibitory factor (CSIF)] is an anti-inflammatory cytokine that is important for maximal Treg function (122). Treg-produced IL-10 suppresses Th17 cells and inhibits IFNγ production by CD4^+^ T cells in inflamed tissues (123, 124). Cell-contact dependent mechanisms include CTLA-4, cell surface TGFβ, and granzyme mediated cell apoptosis (125). CTLA-4 on Tregs down-regulates CD80 and CD86 expression on APC, preventing activation of effector T cells through TCR–MHC–APC interactions (126). Activated Tregs also express GARP-latent TGFβ complex on their cell surface, which may induce FOXP3 expression in activated T cells in areas of inflammation, i.e., infectious tolerance (127).

There has been particular interest in Tregs in the setting of allogeneic HSCT. In murine GvHD models, depletion of CD25^+^ Tregs from BM grafts given to lethally irradiated MHC-mismatched mice significantly increased the severity and mortality from GvHD (128, 129). Conversely, co-transfer of CD4^+^CD25^+^ Tregs with CD4^+^CD25^−^ effector T cells (1:1) from C57BL/6 mice into MHC-mismatched BALB/c mice prevented the lethal GvHD seen with the transfer of CD4^+^CD25^−^ T cells alone (130). More importantly, co-transfer of isolated CD4^+^CD25^+^ Tregs with CD4^+^CD25^−^ conventional T cells into an MHC-mismatched mouse with leukemia were able to prevent GvHD but did not prevent the GvT response (131). Co-transfer of Tregs specific for recipient alloantigens were also able to improve immune reconstitution with faster recovery of total lymphocytes, T, and B cells (132). In human allogeneic HSCT, reduced numbers of CD4^+^CD25^high^, CD4^+^FOXP3^+^, or CD4^+^CD25^high^FOXP3^+^ cells and reduced FOXP3 mRNA have been observed in the PB of patients with GvHD (133–138). Furthermore, Magenau and colleagues demonstrated that Treg frequency at the start of the GvHD reduced linearly with increasing GvHD severity, correlated with the maximum grade of GvHD, and predicted response to treatment (138). In skin and intestinal biopsies, patients with GvHD also have fewer FOXP3^+^ cells per CD8^+^ lymphocyte compared to patients without GvHD or with non-GvHD inflammation (136, 139). Several studies have also demonstrated that the number of CD4^+^FOXP3^+^ Tregs in PBSCH is an independent predictor of acute GvHD in MAC transplants (140, 141). A low graft CD3/Treg ratio in MAC T-replete transplants was also found to be an independent predictor of acute GvHD, NRM, and overall survival (142, 143). Of note, UCB grafts contain CD4^+^CD25^+^ Tregs with the proportion of cells inversely correlating with gestational age up to the levels found in adults (2–5% CD4^+^ T cells) (144). In contrast to adults, the majority of UCB Tregs express naïve markers (CD45RA/CD38), possess a more undifferentiated gene expression profile, and do not show initial suppressor activity upon TCR stimulation (15, 145). However, following antigenic stimulation, these cells upregulate CD25, CTLA-4, and FOXP3, proliferate with a high capacity, and possess potent suppressive activity with high IL-10 production (15, 145). These properties may, therefore, partly explain why UCB transplantation is associated with a lower incidence of GvHD (14).

Using Tregs in allogeneic HSCT may promote immune reconstitution and prevent/treat GvHD while maintaining a GvT response. In recent years, focus has been on the isolation and transfer of Tregs around the time of transplantation. Unfortunately, isolation of human Tregs has been hampered by the lack of Treg-specific surface markers, low Treg frequency, and the limitations of GMP. Most strategies use GMP-grade magnetic bead selection of CD25^high^ cells with the depletion of CD8^+^, CD19^+^, or CD127^high^ cells. These Treg-rich products contain 40–60% CD4^+^CD25^++^FOXP3^+^ T cells with *in vitro* suppressive function (146–148). Using this approach, two clinical trials of adoptive transfer of Tregs have been performed in humans. The first was a Phase I study in which patients with a high-risk of relapse were pre-emptively given up to 5 × 10^6^/kg Tregs prior to donor lymphocyte infusions (DLI) (149). In nine patients, there were no adverse events related to the Tregs. In the second study, 28 HLA-haploidentical transplants were given 2–4 × 10^6^/kg isolated Tregs (50% FOXP3^+^) 4 days prior to receiving CD34^+^ cells and 0.5–2.0 × 10^6^/kg conventional T cells (150). The administration of Tregs into the lymphopenic environment was to allow pre-activation and homeostatic expansion of Tregs *in vivo* (151). Despite the absence of other immunosuppression, only two patients developed grade II–IV acute GvHD. In the context of UCB transplantation, Treg isolation strategies have been problematic due to the lower number of Tregs per CBU. Therefore, *ex vivo* Treg expansion has been necessary. In pre-clinical murine studies, *ex vivo* expanded human UCB CD4^+^CD25^+^ Tregs can prevent allogeneic GvHD, reduce Il-17 production, and tip the Treg/Th17 balance in favor of Tregs (152). Initial expansion protocols of adult Tregs used *in vitro* CD3/CD28 stimulation of Treg-rich isolations in the presence of high dose IL-2 (146, 153). However, small numbers of contaminating cells rapidly expanded in culture, reducing the purity of the final product. In addition, repetitive *in vitro* stimulation was associated with down-regulation of FOXP3 (154). Therefore, several refinements to these protocols have been proposed. First, is the combined use of CD25 and CD45RA to isolate naïve Tregs only and improve Treg purity by depleting contaminating effector cells. Naïve Tregs (CD4^+^CD25^+^CD45RA^+^) are highly proliferative *in vitro* and have stable phenotype and function following culture (146). Second, is the addition of Rapamycin as mTOR inhibition prevents expansion of conventional T cells whilst allowing expansion of Tregs (155–157). Using a combination of Rapamycin, IL-15, and TGFβ, Asanuma and colleagues obtained more than 500-fold expansion of CD4^+^CD25^+^ T cells from UCB and the expanded cells could suppress allogeneic cell cultures by more than 50% (158). Rapamycin may also convert conventional T cells into FOXP3^+^ Tregs. Although the stability of these cells remains contentious, reports suggest that *ex vivo* expansion of Tregs in the presence of Rapamycin and all-trans retinoic acid (ATRA) may be superior to expansion with Rapamycin alone (159). Only Brunstein and colleagues have reported results using expanded human Tregs to prevent GvHD (160). Twenty-three double UCB transplant patients were given expanded Tregs at a dose of 1–30 × 10^5^/kg on day 1, with 13 of these patients receiving an additional dose of 30 × 10^5^/kg on day 15. Tregs were obtained by CD25 bead isolation from third-party UCB and expanded with CD3/CD28 beads and IL-2 for 18 days. There were no reported adverse events from Treg infusion and grade II–IV acute GvHD was reduced compared to 108 historical controls. These studies therefore provide encouraging results and are the first tentative steps toward Treg cellular therapies in UCB transplantation.

While adoptive transfer of human Tregs remains promising, the ultimate goal is to expand Tregs *in vivo* and modification of current GvHD prophylaxis regimens may enable this. Ciclosporin reduces FOXP3 expression in Tregs, significantly reducing *in vitro* and *in vivo* function of allo-stimulated Tregs and Treg expansion (161–163). Conversely, Rapamycin suppresses conventional T cells but does not reduce FOXP3 expression or *in vivo* Treg function (155–157, 164). *In vivo* murine studies have shown that the combination of IL-2 and Rapamycin can selectively expand Tregs and reduce lethal acute GvHD (165). In Phase II clinical studies, the combination of Rapamycin and Tacrolimus has also shown effective prevention of GvHD and lower NRM compared to historical controls (166, 167). However, there are concerns about the increased risk of thrombotic microangiopathies. Novel therapies to increase Tregs *in vivo* are also being investigated. In mice, *in vitro* inhibition of DNA methylation using the hypomethylating agents, Azacitidine or Decitabine, or by knocking out DNA methyltransferase 1 (DNMT-1) induced Foxp3 expression in proliferating naïve CD4^+^CD25^−^ T cells (168–171). Furthermore, Choi and colleagues demonstrated that Decitabine treated naive CD4^+^CD25^−^ T cells from B6 mice (60% Foxp3^+^) were able to protect lethally irradiated BALB/c mice from GvHD when given T-cell depleted BM and conventional T cells from the same B6 mice (171). Using this model, *in vivo* Azacitidine post-transplant (days 15–21) prevented GvHD without inhibiting the GvL response, increased FOXP3^+^ Tregs in the PB, and improved overall survival (171). Azacitidine has also been shown to increase FOXP3 expression in human naive T cells although it has been reported that these cells produce pro-inflammatory cytokines, including IFNγ and TNFα (169, 172). Therefore, it remains unclear whether demethylation agents will increase functional human Tregs post-HSCT and whether this will improve immune reconstitution and/or prevent GvHD. It also remains to be determined whether these agents can be used following UCB transplantation without effecting engraftment. Early phase clinical trials are in process.

### Use of mesenchymal stem cells

Mesenchymal stem cells are multipotent undifferentiated stromal cells with capacity to self renew and/or differentiate into mesenchymal cells including chondrocytes, osteocytes, adipocytes, cardiomyocytes, and neurons. They are present in PB, BM, UCB, and non-hematopoietic tissues including fat, muscle, and UC connective tissue, e.g., Wharton’s jelly, although their exact function *in vivo* remains unclear. MSC are a heterogeneous population that lack hematopoietic markers (CD45/CD34/CD14) but express the antigens SH-3/SH-4 (CD73), Thy-1 (CD90), and Endoglin (CD105) (173). However, there is considerable phenotypic variation between MSC obtained from different sources and there is no universal marker allowing specific isolation of these cells.

In relation to UCB transplantation, MSC have low immunogenicity and potent immunosuppressive function that may be useful for improving engraftment and preventing GvHD. MSC do not express Class II MHC molecules or co-stimulatory molecules and, thus, do not elicit allo-antigenic responses. They can also suppress T and NK-cell proliferation, cytokine secretion, and B cell function (174–176). Possible mechanisms include cell-contact dependent and independent responses including IL-10, TGFβ, nitric oxide, and PGE2 and induction of Tregs (174, 177–179). Pre-clinical murine studies showed that co-transplantation of MSC with UCB CD34^+^ cells in NOD/SCID mice improved engraftment (180–182). In addition, UC MSC supported *ex vivo* expansion of UCB HSC in long-term cultures (183). In 2009, MacMillan and colleagues performed a Phase I/II study of *ex vivo* expanded haploidentical BM-derived MSC in pediatric unrelated UCB transplants (184). Eight patients received MSC [median dose 2.1 × 10^6^/kg (range, 0.9–5.0)] in addition to UCB [median TNC 3.1 × 10^7^/kg (range, 2.0–12.4)], with three patients receiving an additional infusion of MSC on day 21. There were no harmful side effects related to infusion of the MSC. All patients achieved neutrophil engraftment at a median time 19 days (range, 9–28 days). Six patients achieved platelet engraftment at a median of 53 days (range, 36–98 days). Rates of engraftment, GvHD, and survival were comparable to equivalent historical group demonstrating the safety and feasibility of this approach. In a similar pilot study, nine patients received MAC followed by UCB transplants with co-infusion of BM-derived MSC and T-depleted HSC from a third-party donor (185). All patients achieved neutrophil engraftment at a median of 12 days (range, 10–31 days) with full CB chimerism at a median of 51 days (range, 20–186 days). The maximum cumulative incidence of platelet engraftment was 88% (95% CI, 70–100%) at a median of 32 days (range, 13–97 days). However, there was no difference in engraftment rates compared to a control group of 46 transplants from the same center not receiving MSC. In addition, they reported no difference in recovery of lymphocyte populations although their data were not published. Bernardo and colleagues reported similar findings in 13 pediatric UCB transplants using paternal MSC with no difference in engraftment or rates of rejection compared to 39 matched historical controls (186). Recently, a Phase I/II study of UCB transplants with UC-derived MSC has been performed (187). Five patients received *ex vivo* expanded MSC obtained from Wharton’s jelly without any adverse events. Neutrophil engraftment [median 11 days (range, 7–13 days)] and platelet engraftment [median 32 days (range, 22–41 days)] were significantly faster than in nine control patients not receiving MSC. There was no significant difference in total lymphocyte recovery. However, other studies have demonstrated that co-infusion of MSC at the time of UCB transplantation has a negative effect on thymopoiesis and TREC reconstitution, and was associated with reduced survival (188). Therefore, the full implications of co-infusion of MSC with UCB transplantation on engraftment and immune reconstitution require further clarification.

In murine BMT models, MSC have also reduced GvHD, although the timing, dose, and frequency appeared critical as greatest effect was seen when the cells were given into a pro-inflammatory environment with high levels of IFNγ. MSC were first administered to a HSCT patient in 2004 when a 9-year-old boy with refractory GvHD, following a haploidentical transplant, was given BM-derived MSC with complete resolution of symptoms. In 2006, Ringden and colleagues reported a Phase I study in which eight patients with steroid-refractory grade III–IV acute GvHD were given MSC at a median dose of 1 × 10^6^/kg (range, 0.7–9.0) (189). There were no acute effects related to the infusions and six out of eight patients had complete resolution of all symptoms. Overall survival was reported to be better than in a similar group of 16 patients not receiving MSC. In a multi-center EBMT Phase II study, 55 HSCT patients received *ex vivo* expanded BM-derived MSC for steroid-refractory severe acute GvHD (190). Thirty had a complete response and nine had a partial response with median time from infusion to CR of 18 days (range, 3–63 days). Both TRM and overall survival were better in the complete responders. In a multi-center Phase III study, 244 patients with steroid-refractory grade II–IV acute GvHD were randomized to receive third-party MSC (eight infusions of 2 × 10^6^/kg). For liver and gastrointestinal GvHD, the overall response rate at 28 days was higher in the MSC group. However, no significant difference was found in the rate of durable responses (>28 days). MSC have also been used in first-line treatment of GvHD. In a Phase II study, 31 patients with *de novo* grade II–IV acute GvHD were randomized to receive third-party MSC at 2 or 8 × 10^6^/kg in addition to standard corticosteroid therapy. Complete responses were seen in 77% patients and 16% showed a partial response although there was no difference between the low and high dose cohorts. To date, few published studies have examined the use of MSC to treat GvHD following UCB transplantation. In the study by Gonzalo-Daganzo and colleagues using third-party MSC and UCB HSC at the time of transplantation, no difference was observed in acute GvHD (185). However, in this study, two patients who developed steroid-refractory GvHD were subsequently treated with therapeutic infusions of MSC and both had complete resolution of symptoms. In Bernardo and colleagues study, using co-transplantation of UCB and parental MSC, those patients receiving MSC had less grade III–IV acute GvHD (0 vs. 26%, *P* = 0.05) although there was no significant difference for grades II–IV (186). Therefore, the use of MSC to prevent a/or treat GvHD following UCB transplantation may have a role but further investigation is required.

### Use of cytokines, growth factors, and tyrosine kinases

Many cytokines and growth hormones involved in normal thymic function are currently under investigation in pre-clinical studies to determine whether they improve thymopoiesis and immune reconstitution post-HSCT. However, to date, few of these have been specifically tested in the setting of UCB transplantation.

Interleukin-7 is a 25-kDa glycoprotein growth factor produced by the BM stroma and thymic epithelial cells (TEC) and is important for T-cell development. IL-7 binds to its cognate receptor (IL-7R) on immature thymocytes, promoting their differentiation and proliferation into immature T lymphocytes. Mutations of the IL-7 gene or IL-7R produce severe immunodeficiency syndromes (191). In murine allogeneic BMT models, IL-7 increased thymopoiesis and peripheral expansion of recent thymic emigrants and mature T cells, as well as increasing B cells, NK cells, NKT cells, monocytes, and macrophages (192–194). Importantly, in these studies, GvHD was not exacerbated although GvL was maintained (192). However, other similar models have shown increased GvHD using IL-7 (195). In non-BMT human studies, administration of rhIL-7 increases CD4^+^ and CD8^+^ T cells with a preferential expansion of naïve T cells and an increased TCR repertoire (196, 197). More recently, Perales and colleagues reported results from a Phase I trial of rhIL-7 (CYT107) after T-cell depleted allogeneic HSCT (198). Twelve patients were treated with escalating doses of rhIL-7 (10–30 μg/kg). IL-7 produced an increase in effector memory T cells with an associated increase in viral specific T cells and enhanced TCR diversity.

Interleukin-2 is a 15-kDa soluble cytokine produced by activated T cells and is critical for their differentiation and proliferation into effector cells. It also promotes proliferation and expansion of B and NK cells and increases cytotoxic activity and production of effector cytokines. IL-2 binds via its cell surface receptor containing the IL-2Rα subunit (CD25), IL-2Rβ subunit (CD122), and the common gamma chain (CD132). As with IL-7, deficiency in IL-2 or its receptor leads to profound immune dysregulation with chronic infection and severe autoimmunity (199). In HSCT, several groups have shown the IL-2 administered after chemotherapy or DLI may reduce relapse and increase responses in refractory disease, possibly through an enhanced GvL response (200, 201). However, low dose IL-2 produces enhanced Treg proliferation with increased thymic Treg output while having little effect on conventional T cells (202). Therefore, such strategies may improve T-cell homeostasis post-HSCT, preventing GvHD and/or thymic damage. Clinical trials using IL-2 in T-cell depleted double UCB transplants for refractory AML are currently in recruitment (203).

Interleukin-15 is a 14- to 15-kDa glycoprotein belonging to the same family as IL-2 and IL-7. It is expressed by monocytes, macrophages, and dendritic cells and binds to the IL15Rα subunit, IL-2/15Rβ subunit (CD122), and the common gamma chain (CD132). Functionally, it causes proliferation of T cells, B cells, and NK cells and is the primary survival growth factor for NK cells. In murine BMT models, post-transplant IL-15 administration increased donor-derived CD8^+^ T cells, NK cells, and NKT cells with enhanced NK and T-cell function (204). IL-15 was able to enhance the GvL effect but also increased GvHD in T-replete BMT.

Sex steroids have important effects on myeloid and lymphoid recovery following HSCT. The sex steroid hormones (androgen, estrogen) impair thymic function and cause apoptosis in thymic stromal cells and developing thymocytes following puberty. Conversely, reducing sex steroid levels, either via castration or by modifying the hypothalamic–pituitary–gonadal axis, increases thymopoiesis by reducing the rate of apoptosis and increasing the proliferation of TECs. When a luteinizing hormone releasing hormone agonist (LHRHa) is administered in a continuous fashion at high doses, LHRH receptors become desensitized and there is a subsequent reduction in the production of follicle stimulating hormone (FSH) and luteinizing hormone (LH). In turn, this leads to a reduction in the production of sex steroids (205, 206). Using this approach in a murine BMT model produced a significant increase in myeloid and lymphoid progenitors and enhanced thymic reconstitution with increased peripheral T cells (205). GvHD was not increased but GvL was maintained. Furthermore, combined use of keratinocyte growth factor (KGF) and LHRHa increased thymopoiesis with enhanced reconstitution on naïve CD4^+^ and CD8^+^ T cells, reduced homeostatic expansion, and produced a broader T-cell repertoire (207).

Growth hormone (GH) and its mediator insulin-like factor-1 (IGF-1) may also improve thymopoiesis. GH is a 22-kDa protein produced by the pituitary gland, under the control of the hypothalamus via the production of growth hormone releasing hormone (GHRH) and growth hormone inhibiting hormone (GHIH). It is also produced by thymocytes and TEC and acts locally with its corresponding receptor (GHR) in an autocrine fashion. GH induces proliferation and cell growth within the thymus and increases thymic size, cellularity, and TCR repertoire when administered to GH-deficient mice (208). It also accelerates T-cell and immune recovery when given in murine T-depleted HSCT (209). Furthermore, GH may also protect against the effects of radiation, increasing production of HSC and promoting recovery of leukocytes, T, B, and NK cells in murine models (210). IGF-1 is a 7-kDa protein, which is mainly produced by the liver in response to GH but is also produced by other cell types in an autocrine fashion. IGF-1R is expressed on thymocytes, T cells, and TECs and binding of IGF-1 to its receptor increases the proliferation of thymocytes and peripheral T cells (211). Administration of IGF-1 in murine HSCT models also increased *in vivo* thymic precursor populations, donor-derived T cells as well as pro-, pre-, and mature B cells, and myeloid cells (212).

Keratinocyte growth factor is a 28-kDa protein produced by mesenchymal stromal cells and mature thymocytes. Several groups have shown that KGF protects mucosal, cutaneous, and epithelial cells from cytotoxic and irradiation induced injury (213, 214). In murine BMT models, pre-treatment with KGF protected the TECs and increased donor-derived thymocytes, peripheral naïve T cells, and production of IL-7 (215). KGF induces p53 and NF-kappa pathways in immature TECs, promoting proliferation and differentiation into mature TECs (216). In a murine UCB transplant model, KGF pre-treatment increased day 35 thymic outputs with higher T-cell and NKT-cell numbers within the spleen and increased the proportion of TREC (217). However, in human clinical trials, although KGF (Palifermin) reduced mucositis, it has not been shown to have any significant impact on lymphocyte reconstitution, GvHD, infectious complications, or overall survival (218–221).

Finally, other cellular pathways currently being investigated to improve thymopoiesis post-HSCT are the tyrosine kinases, Flt-3 and c-Kit, and the tumor suppressor gene, p53. FLT-3-L, produced in the thymus and expressed on the surface of perivascular fibroblasts, is upregulated following irradiation and increases proliferation of Flt-3 positive thymic precursors (222). Flt-3-L also promotes thymocyte maturation and homeostatic expansion of peripheral T cells following HSCT (223). Stem cells factor (SCF) also promotes early thymocyte development through its receptor tyrosine kinase, c-Kit. Pre-clinical studies in mice using the tyrosine kinase inhibitor, Sunitinib, suggest that pre-treatment of the recipient may promote improved donor-derived thymopoiesis by blocking c-Kit in the host’s early thymic progenitors and, thus, improving accessibility to the thymic niche (224). p53 is a tumor suppressor gene that activates DNA repair, arrests cell growth, and induces apoptosis in response to cell damage. Given the degree of epithelial damage following conditioning chemotherapy and/or irradiation, it has been proposed that temporary inhibition of p53 may reduce thymic apoptosis and promote T-cell recovery following HSCT. In murine HSCT models, Kelly and colleagues demonstrated that temporary inhibition of p53 using the small molecule pifithrin-β (PFT-β) prevented damage in TECs (225). Moreover, when combined with KGF, thymic function was improved post-HSCT with higher numbers of donor-derived naïve CD4^+^ and CD8^+^ T cells and enhanced responses against *Listeria monocytogenes*.

## Conclusion

The scientific disciplines of immunology, cellular biology, and immune regulation following UCB transplantation continue to rapidly advance and over recent years, our understanding of how cord blood and neonatal immune cells differ from those found in adults has become clearer. This information is essential to improve our understanding of immune reconstitution, and perhaps, the means to accelerate recovery after UCB transplantation. Despite our extensive knowledge on the unique biology of CB graft lymphocytes, many of the characteristics of these cells and their relevance to immune cell recovery have still not been adequately evaluated after UCB transplantation. Therefore, more in depth pre-clinical and clinical studies in these areas are warranted, both in terms of recovery of normal immune cell function and their effectiveness in antitumor cell activity.

## Conflict of Interest Statement

The authors declare that the research was conducted in the absence of any commercial or financial relationships that could be construed as a potential conflict of interest.
